# The Conserved Lysine-265 Allosterically Modulates Nucleotide- and Actin-binding Site Coupling in Myosin-2

**DOI:** 10.1038/s41598-017-07933-y

**Published:** 2017-08-09

**Authors:** Vincent A. Behrens, Stefan Münnich, Georg Adler-Gunzelmann, Claudia Thiel, Arnon Henn, Sharissa L. Latham, Manuel H. Taft

**Affiliations:** 1 Institute for Biophysical Chemistry, Hannover Medical School, OE 4350, Carl-Neuberg-Str. 1, 30625 Hannover, Germany; 2 Faculty of Biology, Technion - Israel Institute of Technology, Haifa, 3200003 Israel; 3 Institute for Molecular- and Cell Physiology, Hannover Medical School, Hannover, Germany; 4 Department of Structural Biology, Stanford University, 299 Campus Drive, 94306 Stanford, California United States

## Abstract

Myosin motor proteins convert chemical energy into force and movement through their interactions with nucleotide and filamentous actin (F-actin). The evolutionarily conserved lysine-265 (K265) of the myosin-2 motor from *Dictyostelium discoideum* (*Dd*) is proposed to be a key residue in an allosteric communication pathway that mediates actin-nucleotide coupling. To better understand the role of K265, point mutations were introduced within the *Dd* myosin-2 M765-2R framework, replacing this lysine with alanine (K265A), glutamic acid (K265E) or glutamine (K265Q), and the functional and kinetic properties of the resulting myosin motors were assessed. The alanine and glutamic acid substitutions reduced actin-activated ATPase activity, slowed the *in vitro* sliding velocity and attenuated the inhibitory potential of the allosteric myosin inhibitor pentabromopseudilin (PBP). However, glutamine substitution did not substantially change these parameters. Structural modelling suggests that K265 interacts with D590 and Q633 to establish a pivotal allosteric branching point. Based on our results, we propose: (1) that the K265-D590 interaction functions to reduce myosins basal ATPase activity in the absence of F-actin, and (2) that the dynamic formation of the K265-Q633 salt bridge upon actin cleft closure regulates the activation of product release by actin filaments.

## Introduction

Myosins generate force and movement through their ATP dependent interaction with actin. Isoforms within this superfamily of molecular motors regulate a multitude of physiological processes in both muscle and non-muscle cells, including sarcomere assembly and contractility, cell motility and division, intracellular vesicle transport and docking, and membrane blebbing. As such, there is great interest in identifying and synthesising compounds that can specifically activate or inhibit select myosin isoforms. Effectors of allosteric communication pathways, such as Omecamtiv Mecarbil^1, 2^, EMD 57033 derivatives^3^, Ammosamides^4, 5^, Blebbistatin derivatives^6–8^ and halogenated Pseudilins^9–11^, thus far show the greatest promise for achieving this isoform specificity.

Studies assessing the inhibitory effect of halogenated Pseudilins on myosin-1, -2 and -5 motors identified an allosteric communication pathway connecting residues in the actin binding region with those in the active site^9–11^. It was postulated that these compounds were effective in part via their interaction with an evolutionarily conserved lysine residue, which is located 16 Å from the active site, at position 265 (K265) within the *Dd* motor domain. This importance of K265 is further highlighted in recent studies examining the inhibitory effects of *Fusarium*-specific cyanoacrylate fungicides on *Fusarium* myosin-1 and myosin-5^12–14^. The cyanoacrylate fungicides are predicted to bind in the same allosteric pocket as PBP^14^. The *Fusarium* strains that were resistant to the compound had mutations at the residue corresponding to K265 in *Dd* (K216Q in myosin-1 of the resistant *F. avenaceum* 05001 strain and K216E in myosin-5 of the resistant *F. graminearum* Y2021B).

Accordingly, this study sought to characterize the kinetic and structural basis for the K265-mediated modulation of myosin motor function. We made use of the *Dd* myosin-2 M765-2R construct^15, 16^ as a model system for enzymatic and functional studies. This construct, which consists of the *Dd* myosin-2 motor domain and two *Dd* α-actinin repeats, permits the kinetic and functional properties of the core motor domain to be examined in the absence of light-chain regulation, in turn simplifying both protein production and data interpretation^15–22^. To define the molecular role of this conserved amino acid in the proposed allosteric pathway, we generated mutant constructs where the lysine at position 265 is substituted for alanine (K265A), glutamic acid (K265E) or glutamine (K265Q). These substitutions, which effectively remove a side chain (K265A), reverse the charge of the amino acid (K265E) or remove the charge and replace the amino acid with one of a similar size (K265Q), give insights regarding the immediate interactions of K265. We performed steady-state and transient enzyme kinetics, as well as direct functional assays to investigate the molecular mechanism by which these mutations affect the communication between nucleotide- and actin-binding sites. Based on previously published crystal structures of the myosin motor domain^23–26^, we suggest that K265 is located at a branching point in the allosteric pathway, where its interaction with Q633 at the base of loop-2 supports strong actin-binding and the actin-mediated activation of product release, and thereby myosin ATPase activity. In addition, the interaction of K265 with D590 in the strut loop appears to attenuate the basal ATPase in the absence of actin and to modulate nucleotide-binding properties of the myosin motor domain.

## Results

### Protein Expression and Purification

M765-2R wild type (WT) and the mutant constructs were over-produced in axenic *Dd* cell cultures and purified to homogeneity. The majority of constructs gave yields of 3.3 to 6.7 mg/L of culture medium. Approximately 6-fold lower yields were obtained with construct K265A. Moreover, active-site titrations with ATP showed only ~60% of the purified K265A construct to be enzymatically competent. The fraction of enzymatically active protein was greater than 80% for M765-2R and mutant constructs K265E and K265Q.

### Functional properties of myosin constructs as analysed by *in vitro* motility assays

**Figure 1:**
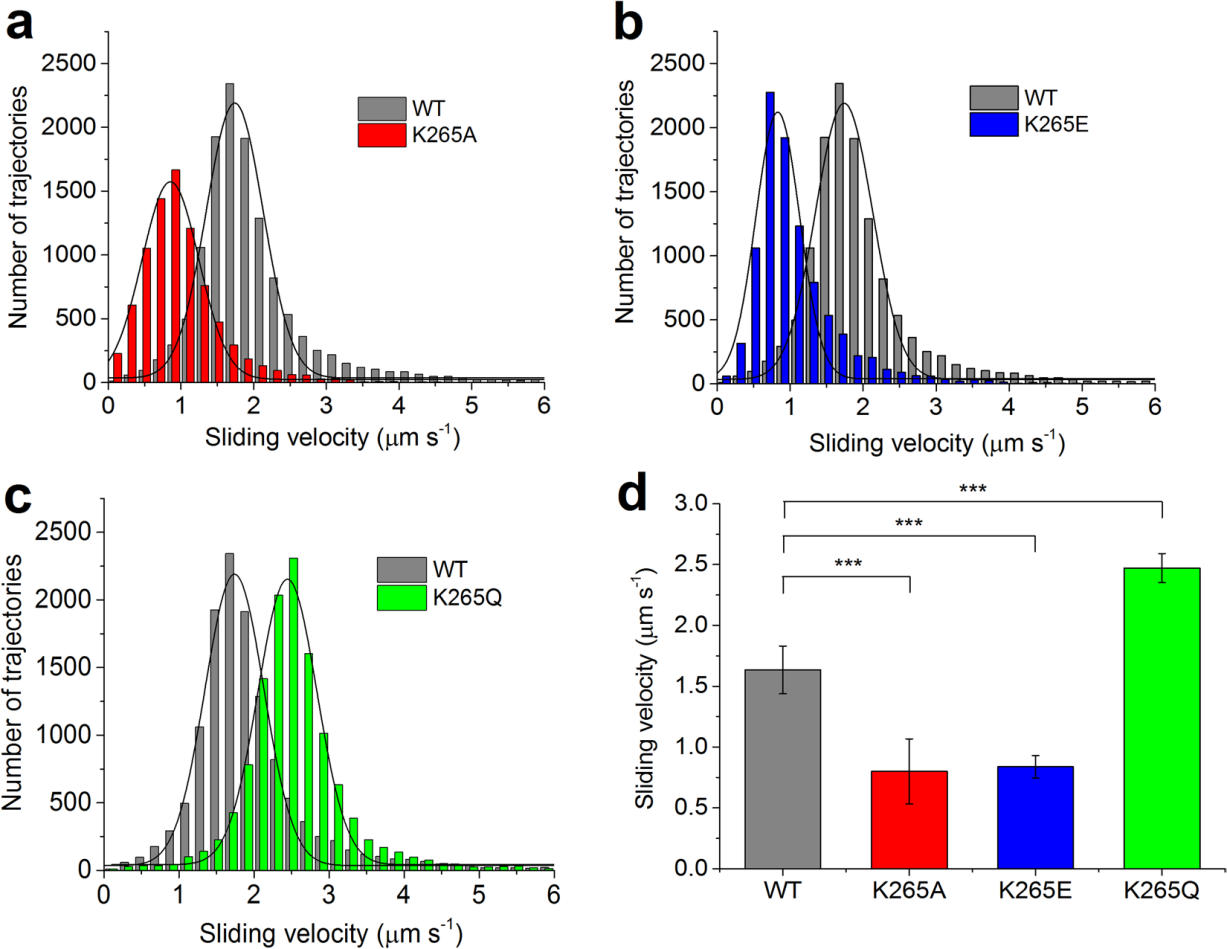
Sliding velocity of actin filaments on WT and mutant myosin-decorated surfaces as determined by *in vitro* motility assays. (**a**–**c**) The histograms and Gaussian fits show typical distributions and average sliding velocities of actin filaments on lawns of WT compared to K265A, K265E and K265Q myosin lawns. (**d**) The bar graph represents the mean values of 7–10 different experiments with one or two protein preparations; the error bars show the standard deviation. The differences in sliding velocity obtained for the mutants were significant as compared to WT (*** for p < 0.0005).

We first wanted to assess the functional competence of the myosin constructs used in this study, and thus performed *in vitro* motility assays with all four constructs. We tracked the sliding of more than one hundred actin filaments per experiment to obtain individual sliding velocity distributions that could be fit with a Gaussian function in order to calculate the average actin filament sliding velocity (Fig. 1a-c). We found that the average sliding velocity of K265A and K265E mutants is more than 2-fold slower (0.80 ± 0.27 µm s^−1^ and 0.84 ± 0.09 µm s^−1^) compared to the WT (1.63 ± 0.19 µm s^−1^), whereas the K265Q mutant showed a faster sliding velocity (2.47 ± 0.12 µm s^−1^) (Fig. 1d).

### Basal ATPase activity and basal Pi-release of WT and mutant myosins

**Figure 2:**
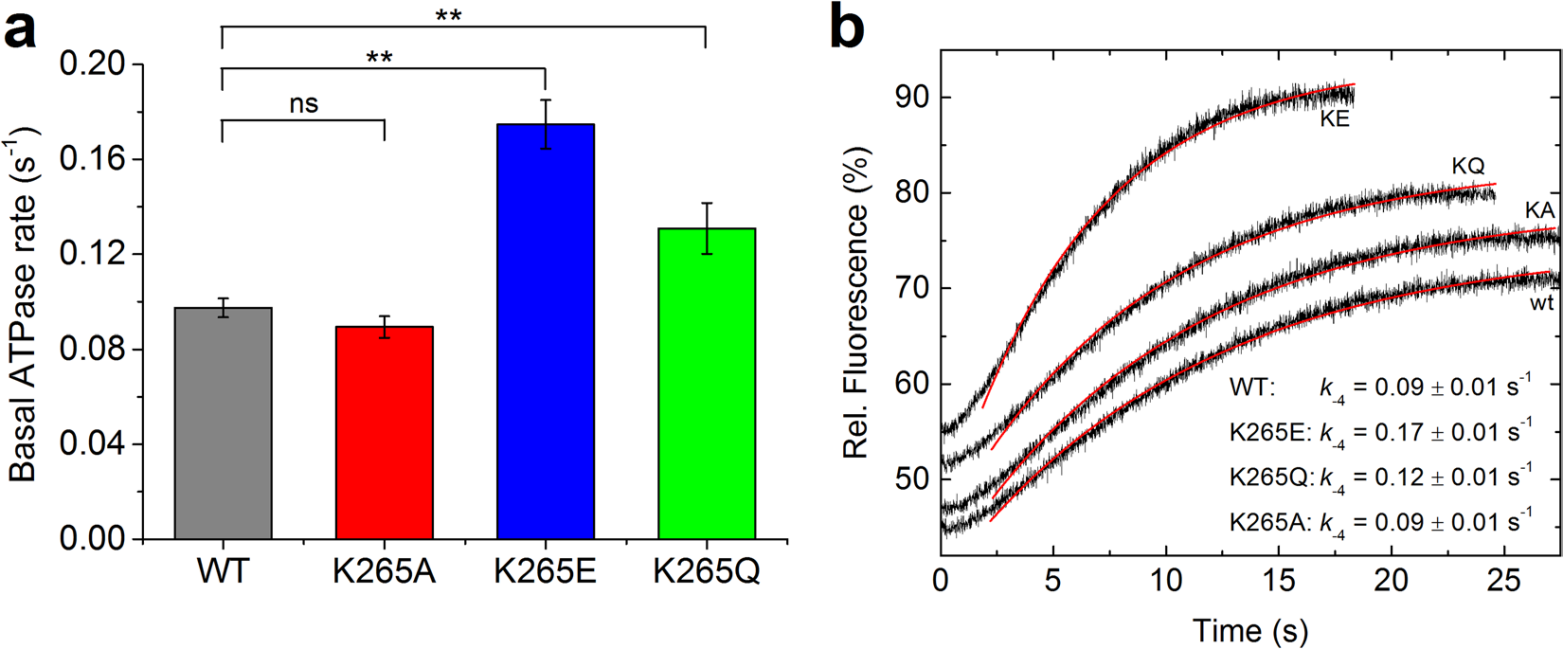
Basal ATPase activity and basal P_i_-release of WT and mutant myosins. (**a**) The basal ATPase rate was determined in the absence of actin. Error bars give the standard deviation for 3–6 independent experiments. The differences obtained for K265E and K265Q were highly significant (** for p < 0.005). (**b**) The basal P_i_-release rate *k*_-4_ is the rate limiting step of the basal *Dd* myosin-2 ATPase cycle and was measured using a fluorescent P_i_ detection system. The obtained kinetic parameters are summarized in Table 1(a) and Table 2(b).

**Figure 3:**
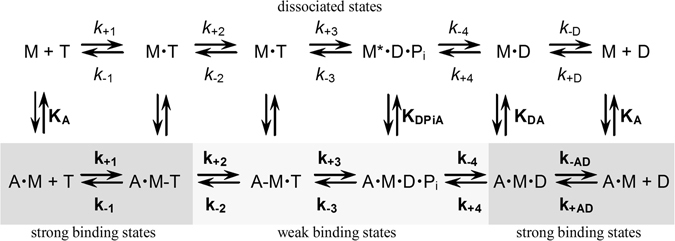
Interaction scheme for actin and nucleotide binding of myosin. A refers to actin, M to myosin, T to ATP, D to ADP and Pi to inorganic phosphate. The asterisk (*) indicates the increased fluorescence of W501 induced by ATP hydrolysis. For the equilibrium binding constants a notation is used that distinguishes between constants in the absence and presence of actin by using italics type (*k*_x_, *K*_x_) and bold (**k**_**x**_, **K**_**x**_), respectively.

The basal Mg^2+^-ATPase activity (*k*_basal_) of the myosin-2 WT and mutant proteins was assessed. The WT M765-2R protein displayed a basal ATPase rate of 0.097 ± 0.004 s^−1^, consistent with previously reported values^15^, which was unchanged when the lysine was substituted for alanine (Fig. 2a, Table 1). However, the K265E and K265Q mutants displayed 80% and 35% increases in their basal Mg^2+^-ATPase activities, respectively (p < 0.005 for both, Fig. 2a and Table 1). Although the basal ATPase activity is of less physiological relevance as myosin’s ATPase rate is strongly accelerated by actin, assessing this parameter provides us with mechanistic information about the complete ATPase cycle including all kinetic states (cf. Fig. 3).

**Table 1:** Summary of the steady state ATPase parameters for myosin-2 WT and mutant constructs.

constant	*Dd* myosin-2 (M765-2R construct)			
	WT	K265A	K265E	K265Q
*k*_basal_ (s^−1^)	0.097 ± 0.004	0.089 ± 0.004	0.175 ± 0.010	0.131 ± 0.011
*k*_cat_ (s^−1^)	2.51 ± 0.38	1.18 ± 0.10	0.72 ± 0.12	4.40 ± 0.70
*K*_app_ (μM)	83 ± 21	60 ± 10	47 ± 17	230 ± 50
*k*_cat_/*K*_app_ (μM^−1^s^−1^)*	0.030 ± 0.016	0.020 ± 0.006	0.015 ± 0.013	0.019 ± 0.009
*k*_cat_/*K*_app_ (μM^−1^s^−1^)**	0.035 ± 0.009	0.016 ± 0.001	0.010 ± 0.001	0.021 ± 0.001
Activation***	25.9	13.3	4.11	33.6

Experimental conditions: 25 mM HEPES, 25 mM KCl, 5 mM MgCl_2_, 1 mM DTT, 1 mM ATP, pH 7.3; T = 25 °C.

*calculated from *k*_cat_ and *K*_app_.

**slope of the first four values of actin-activated ATPase measurements.

***calculated as *k*_cat_/*k*_basal_.

As P_i_-release is the rate-limiting step in the ATPase cycle of WT *Dd* myosin-2, we further investigated the changes in the basal ATPase rates by measuring P_i_-release in the absence of F-actin (Fig. 2b). Compared to WT, the rate constant *k*_−4_ (cf. Fig. 3) does not change for K265A, but is increased by 88% and 33% for K265E and K265Q, respectively. These absolute values for the P_i_-release rates are consistent with the basal ATPase rates.

### Actin-activated ATPase activity of WT and mutant myosins

**Figure 4:**
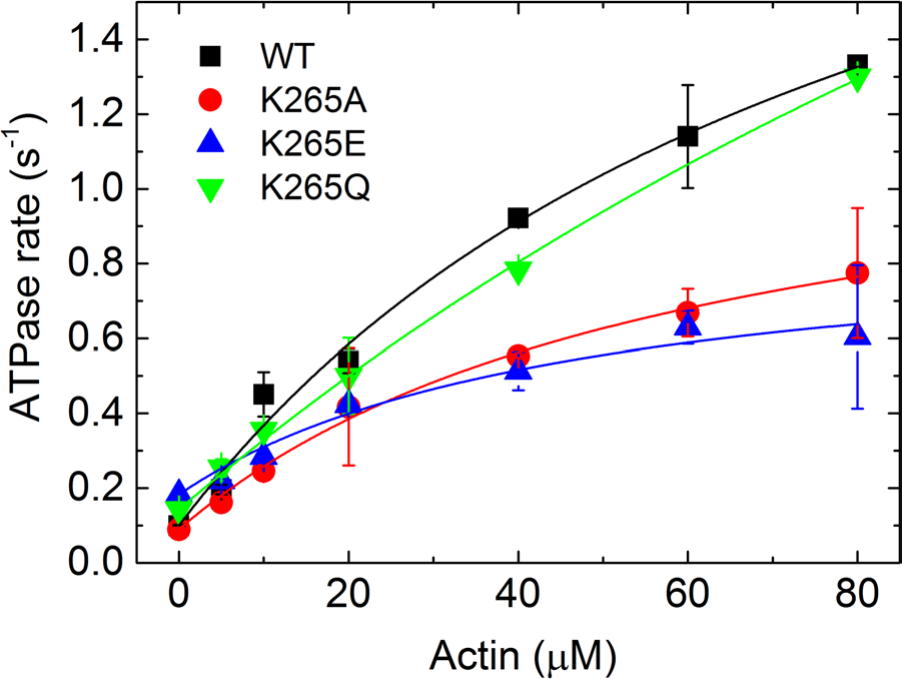
Steady-state ATPase activity of WT and mutant constructs. Determination of the ATPase activity for the WT and the three mutants from 0 to 80 µM F-actin. Data are mean values of 3–6 independent measurements and error bars are the standard deviation. Constants derived from this experiment are summarized in Table 1.

The steady-state Mg^2+^-ATPase activity for all four constructs was measured as a function of F-actin (Fig. 4, Table 1). All mutant constructs displayed active turnover of ATP in steady-state ATPase assays. The hyperbolic fit to the data gave a maximum value for the ATPase activity (*k*_*cat*_) and the actin concentration at half maximum activation (*K*_*app*_). All three mutant constructs show differences in their maximum actin-activated ATPase activities, as well as in their *K*_*app*_ values compared to the WT. For K265A and K265E mutants, the *k*_*cat*_ is reduced by 47% and 71%, respectively, compared to the WT myosin. In contrast, the K265Q mutant has a nearly 2-fold higher *k*_*cat*_ when compared to WT. Whilst the *K*_*app*_ of K265A and K265E is 30% and 40% decreased, respectively. The *K*_*app*_ for K265Q appears to be 3-fold higher than that of the WT. As the fitted values for *k*_*cat*_ and *K*_*app*_ are not well defined for the K265Q mutant because saturation is not reached, we used an alternative approach to rationalize the activation of the myosin ATPase activity by actin: we applied linear fits to fit the data at actin concentrations well below the *K*_*app*_, and the apparent second-order rate constant for F-actin binding (*k*_cat_/*K*_app_) could then be determined from the slopes of these fits. All mutants display a reduced *k*_cat_/*K*_app_ in comparison to WT. For K265A it is 54% lower, for K265E 71% and for K265Q 40% (Table 1). We then compared the ATPase activation by F-actin from the ratio of *k*_*cat*_/*k*_*basal*_. While the WT is activated 26-fold, K265A is activated only 13-fold and K265E displays an even lower 4-fold activation. Interestingly, the K265Q mutation appears to recover WT function as the activation is even slightly increased to about 34-fold.

### Binding and release of F-actin

**Figure 5:**
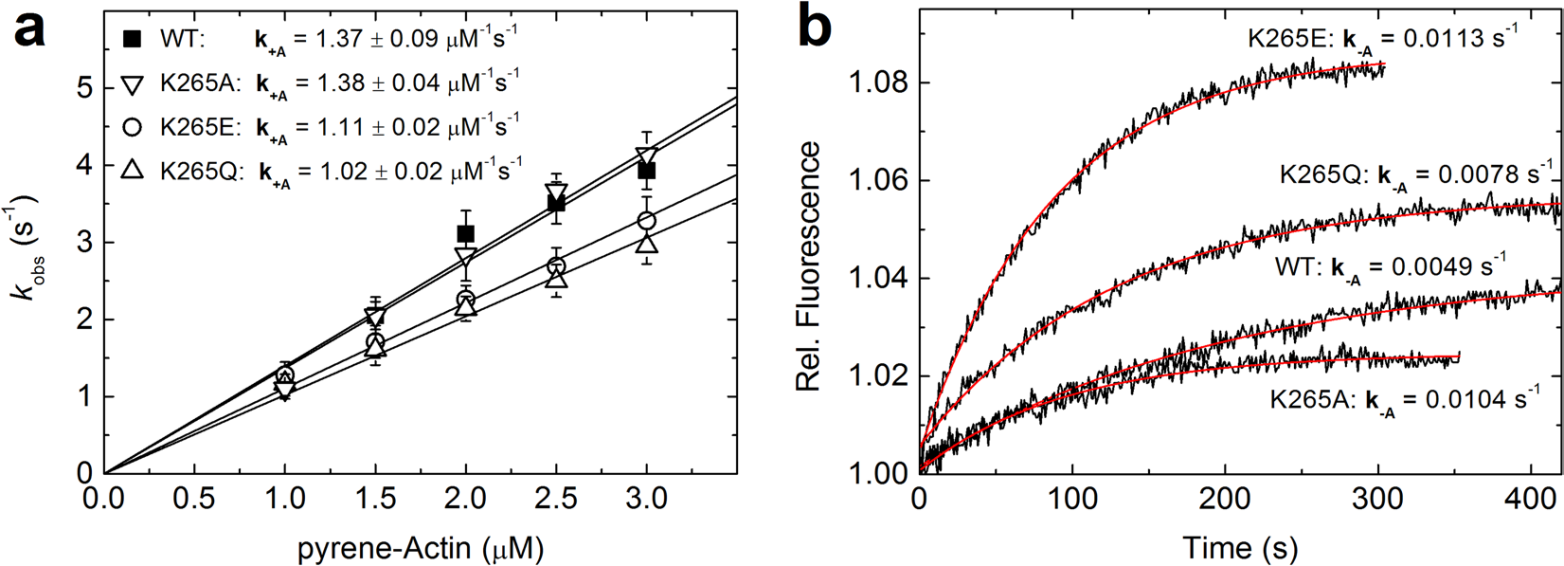
Binding and release of F-actin. Interaction of F-actin with WT and mutant myosin was followed using pyrene-labelled F-actin. (**a**) The actin concentration-dependent increase in the observed rate constants for pyrene-actin binding could be fit by a linear function. The slope defines the actin binding rate **k**_**+A**_, which is slightly decreased by mutations K265E and K265Q. Error bars give the standard deviation for three independent experiments. (**b**) Fluorescence transients observed after chasing pyrene-actin from the complex with myosin by excess unlabelled F-actin. Single exponential fits to the data define F-actin release rate constants, which are significantly increased for all three mutant myosins. All resulting kinetic parameters for F-actin binding and release are summarized in Table 2.

The binding of pyrene-actin to myosin was monitored by the decrease in pyrene-fluorescence that accompanies the formation of the actomyosin complex (Fig. 5a). The fluorescence transients could be described by single-exponential fits and the respective rate constants were plotted against the actin concentration. Linear fits to the data yielded second order binding rate constants for actomyosin complex formation **k**_**+A**_, which are decreased for the K265A and the K265E construct by 19% and 26%, respectively. The K265Q construct displayed similar binding kinetics as the WT construct. The dissociation of F-actin from myosin (**k**_**−A**_) was determined by competing the bound pyrene-actin with a large excess of unlabelled F-actin (Fig. 5b). The resulting increase in the pyrene-fluorescence signal exhibited mono-phasic behaviour and therefore was fitted by a single exponential function. All mutant constructs showed enhancement in F-actin release rate, which was more than 2-fold accelerated for the K265A and the K265E mutants, and 60% increased in the case of K265Q.

The dissociation equilibrium constant (**K**_**A**_) for F-actin binding to different mutant constructs was calculated from the ratio **k**_**-A**_/**k**_**+A**_. Our results show approximately 2-fold reduced actin affinities for K265A and K265Q and a 3-fold reduction for K265E.

### ATP binding to myosin

**Figure 6:**
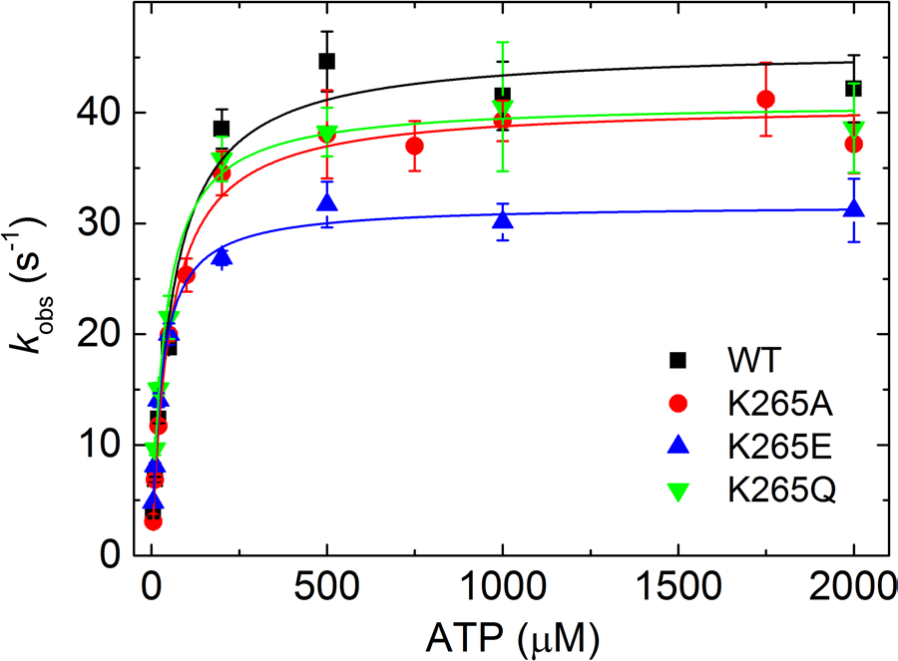
Determination of ATP binding and hydrolysis rates for WT and mutant myosin constructs. The binding and subsequent hydrolysis of ATP by *Dd* myosin-2 induces conformational changes according to Fig. 3, which can be detected by a change of myosin’s intrinsic tryptophan fluorescence. The rate of ATP hydrolysis (*k*_+3_ + *k*_−3_) was decreased with high significance (p < 0.005) for K265E (see Table 2).

We followed ATP binding to the myosin motor domain by the subsequent increase in intrinsic tryptophan fluorescence (Fig. 6, Table 2). Measurements from 0 to 2000 µM ATP were performed and the resulting fluorescence change over time was fitted by single exponentials to obtain ATP binding rate constants *k*_*obs*_. We found a linear dependence of the observed rate constants from 0 to 20 µM ATP. By linear fitting of these values, *K*_1_ *k*_+2_ was determined from the slope. Only minor changes in *K*_1_ *k*_+2_ were observed for the mutants compared to WT. At 500 µM ATP the observed rate constants reached a plateau. Here, a highly significant decrease (30%, p < 0.005) in the rate of ATP hydrolysis (*k*_*+*3_ + *k*_*−*3_) was observed for the K265E mutant compared to the WT. The hydrolysis rates of K265A and K265Q are comparable to the WT. The apparent ATP affinity (1/*K*_1_) of K265E is 50% higher than that of the WT and the other two mutants. From *K*_1_ *k*_+2_ and 1/*K*_1_ the rate constant *k*_*+2*_ was calculated and found to be decreased by 47% and 38% for K265E and K265Q, respectively.

**Table 2:** Summary of the rate and equilibrium constants of the interactions of the myosin-2 WT and mutant constructs with nucleotides and actin.

Parameter (unit)	*Dd* myosin-2 (M765-2R construct)			
	WT	K265A	K265E	K265Q
*K*_1_ *k*_+2_ (µM^−1^s^−1^)	0.58 ± 0.03	0.56 ± 0.07	0.61 ± 0.01	0.54 ± 0.01
*k*_+3+_ *k*_−3_ (s^−1^)	46 ± 1.7	41 ± 0.8	32 ± 0.5	41 ± 0.9
1/*K*_1_ (µM)	56 ± 10	51 ± 5	28 ± 2	37 ± 4
*k*_+2_ (s^−1^) ^$^	32.5	28.6	17.1	20.0
*k*_−4_	0.09 ± 0.01	0.09 ± 0.01	0.17 ± 0.01	0.12 ± 0.01
*k*_−4_^#^	0.07 ± 0.03	n.d.	0.17 ± 0.01	n.d.
*k*_-D_ (s^−1^)	1.82 ± 0.03	1.64 ± 0.06	2.11 ± 0.25	2.61 ± 0.17
**K**_**1**_ **k**_**+2**_ (µM^−1^s^−1^)	0.16 ± 0.01	n.d.	0.15 ± 0.01	n.d
**k**_**+2**_ (s^−1^)	1350 ± 118	1037 ± 326	1150 ± 155	1147 ± 190
1/**K**_**1**_ (µM)	7130 ± 942	4474 ± 2326	5390 ± 1120	5641 ± 1432
**k**_**+A**_ (µM^−1^s^−1^)	1.37 ± 0.05	1.38 ± 0.04	1.11 ± 0.02	1.02 ± 0.02
**k**_**-A**_ (s^−1^)	0.0049 ± 0.0004	0.0104 ± 0.0008	0.0113 ± 0.0009	0.0078 ± 0.0007
**K**_**A**_ (nM)	3.6 ± 0.5	7.5 ± 0.7	10.1 ± 0.9	7.6 ± 0.9
**K**_**DPiA**_ **k**_**−4**_ (µM^−1^s^−1^)	0.034 ± 0.003	n.d.	0.013 ± 0.001	n.d.
**k**_**−4**_ (s^−1^)^*^	>0.95 ± 0.08	n.d.	>0.51 ± 0.05	n.d.
**k**_**-AD**_ (s^−1^)	47 ± 4	n.d.	54 ± 3	n.d.
**K**_**AD**_ (µM)	73.3 ± 7	n.d	40.7 ± 8	n.d

Experimental conditions: 25 mM MOPS, 100 mM KCl, 5 mM MgCl_2_, 1 mM DTT, pH 7.0; T = 20 °C.

^$^calculated ^#^ from y-intercept of actin titration^*^ at 25 µM F-actin with 25 mM KCl.

### ATP-induced dissociation of actomyosin

**Figure 7:**
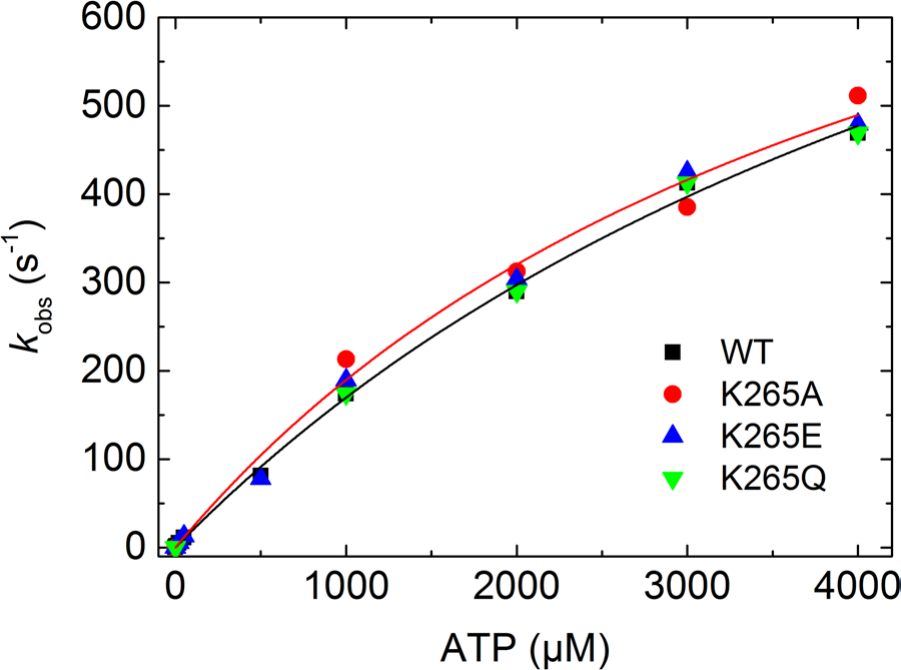
ATP-induced dissociation of acto-myosin complexes for WT and mutant myosin constructs. The dissociation of the rigor acto-myosin complex by increasing concentrations of ATP was followed by the exponential decrease in the light scattering signal. The plot of the observed rate constants versus the ATP concentration was fit by a hyperbola. For clarity, only the fits for WT and K265A are shown (see Table 2).

The dissociation of the actomyosin complex was monitored by the reduction in light scattering after addition of ATP, which followed single exponential kinetics. A plot of the observed rate constants against the ATP concentration was fitted by a hyperbola to give the maximum dissociation rate **k**_**+2**_ and the apparent ATP affinity **1/K**_**1**_ (Fig. 7). A linear fit to the data below 1000 µM ATP yielded the second order rate binding constant of ATP to actomyosin (**K**_**1**_ **k**_**+2**_). Our experiments indicate that none of these parameters were changed significantly for the mutants compared to WT (Table 2).

### Actin-activated Pi-release of WT and mutant myosins

**Figure 8:**
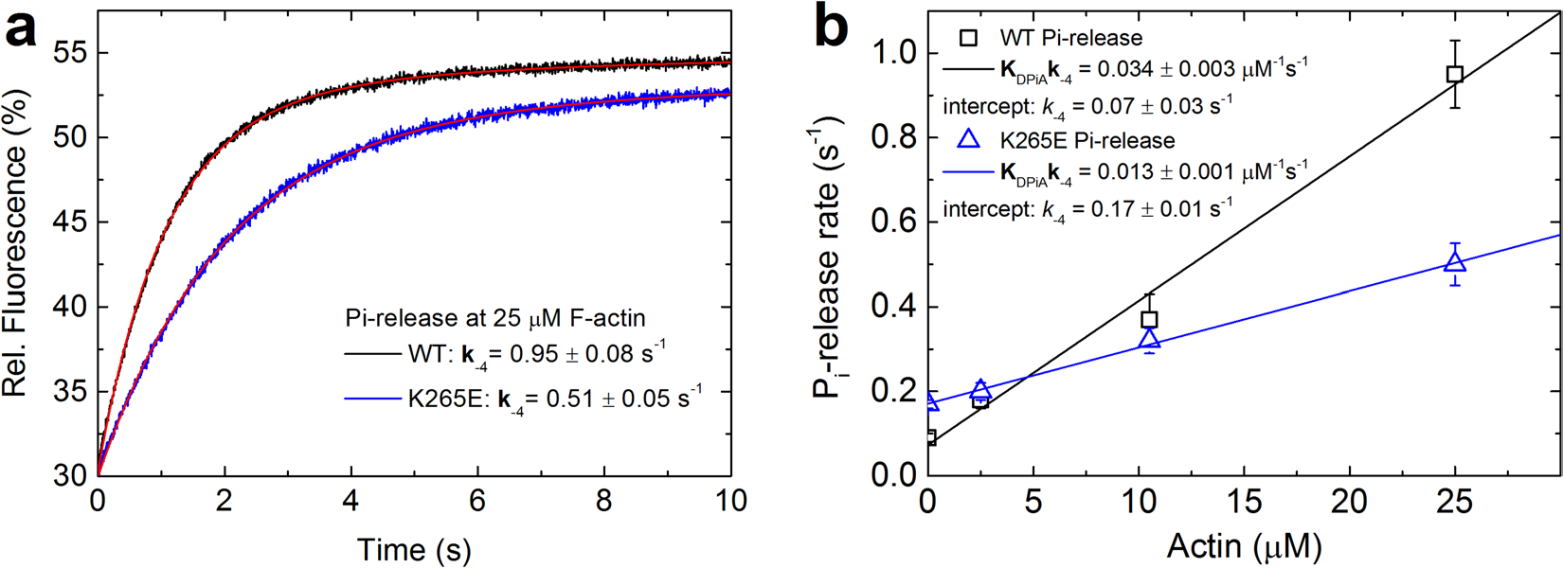
Actin activation of P_i_-release for WT myosin and K265E mutant. (**a**) Averaged fluorescence traces for P_i_-release at 25 µM F-actin are shown for the WT and K265E. (**b**) The P_i_-release was determined in the presence of F-actin in a range of 0 to 25 µM. Error bars report the standard deviation and rate constants are listed in Table 2.

The K265E mutant showed the most pronounced effects on functional competence and ATPase activity. Therefore, we decided to determine actin activation of the P_i_-release step for the WT and K265E constructs in the presence of 0 to 25 µM F-actin. The results show that the actin activation of P_i_-release is clearly lower for the K265E construct (Fig. 8a,b). This is consistent with the steady state measurements, whereby the basal ATPase rate was increased, but the actin-mediated activation was strongly reduced for K265E (Fig. 4). It should be noted that whilst we performed the full, four-point actin titration only with the K265E mutant, the basal (Fig. 2) and actin-activated P_i_-release rates of the other mutants appeared to follow a similar trend as they also resembled the respective ATPase rates.

### ADP release from myosin

**Figure 9:**
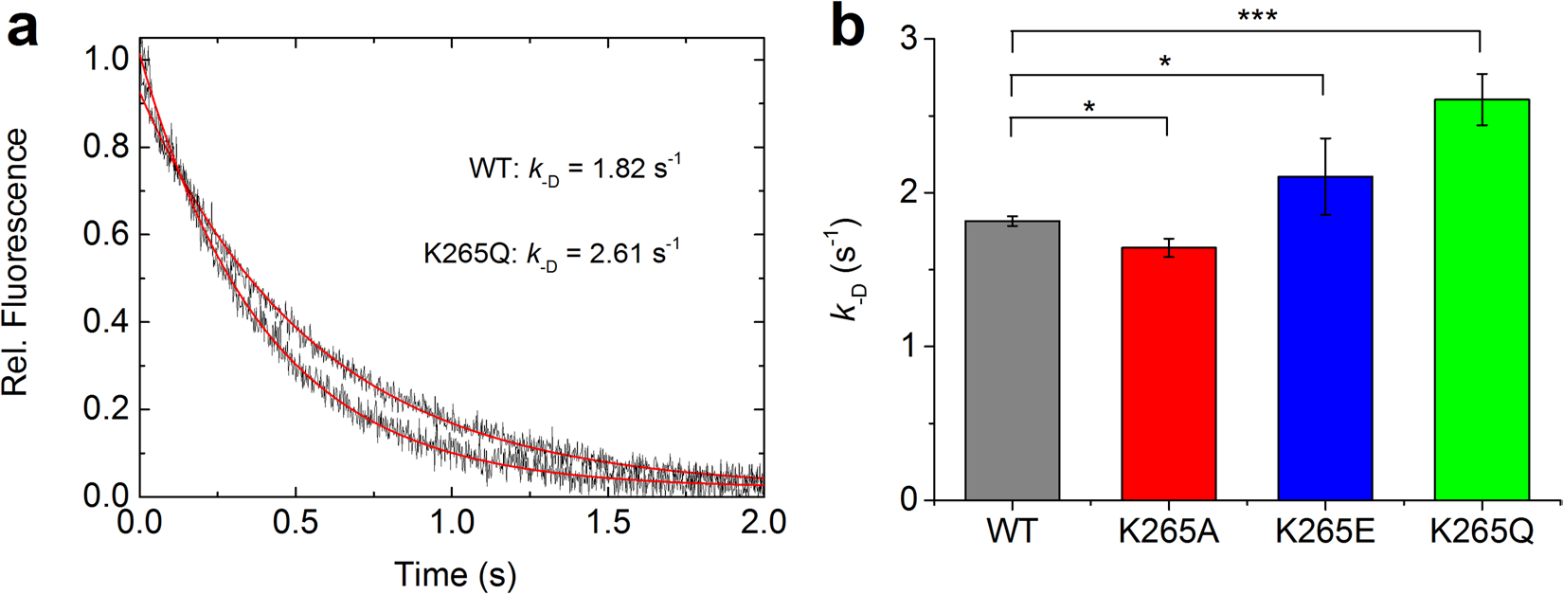
ADP release from myosin. (**a**) Averaged fluorescence traces of mant-ADP release after addition of excess (1 mM) ATP to a complex of 1 µM myosin and 20 µM mant-ADP (for clarity, only WT and K265Q data are shown). (**b**) The bar graph shows the ADP release rates and statistical significance for all four constructs. Error bars report the standard deviation and rate constants are listed in Table 2.

The fluorescence decrease that is associated with the displacement of mant-ADP (2’/3’-O-(N-Methyl-anthraniloyl)-ADP) from the nucleotide binding site by ATP was used to monitor the rate of ADP release from myosin in the absence of actin (Fig. 9). The dissociation constant (*k*_-D_) obtained from these data indicate that there is a significant decrease in the ADP release rate for the K265A construct (10% reduction, p < 0.05), whilst the K265E protein displayed an increase of 16% (p < 0.05). The K265Q construct exhibits the largest increase in *k*_-D_, with by 43% (p < 0.0005) (Table 2).

### ADP inhibition of ATP-induced dissociation of actomyosin

**Figure 10:**
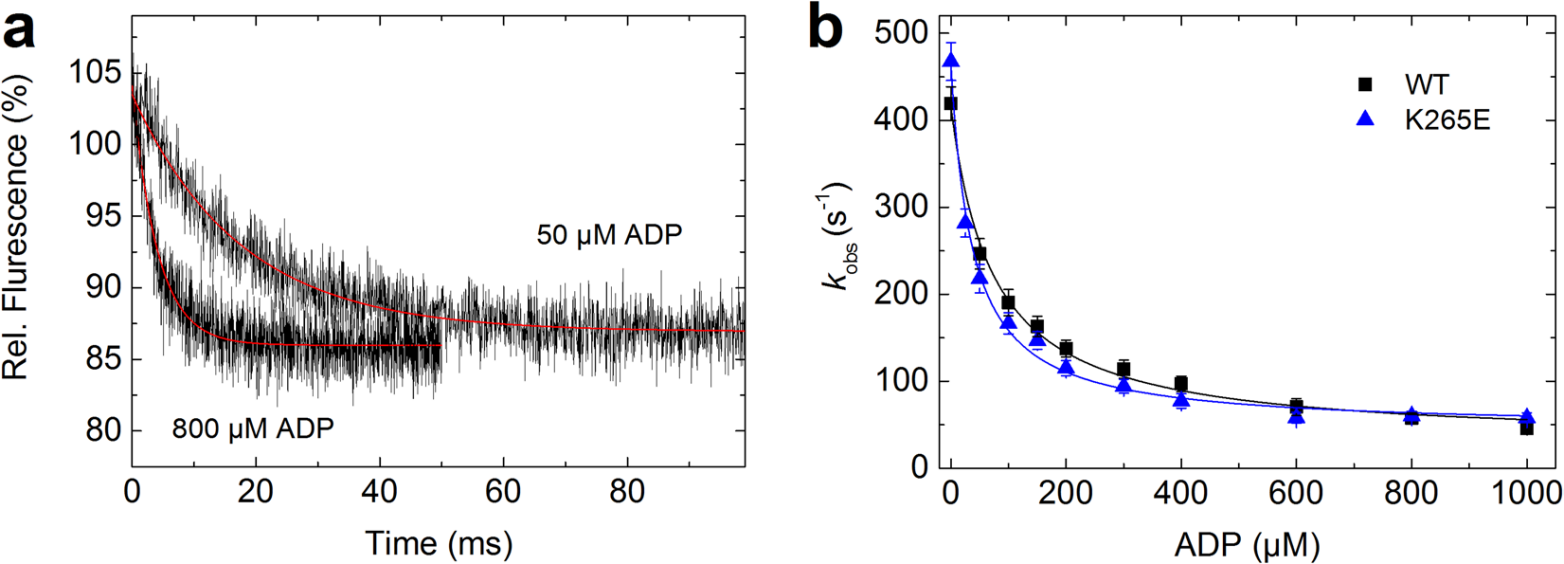
ADP inhibition of ATP-induced dissociation of acto-myosin complexes for WT and K265E. (**a**) The inhibition of the ATP-induced dissociation of the acto-myosin complex was followed by the exponential decrease in the light scattering signal. The graph shows sample traces for the WT at 50 and 800 µM ADP. (**b**) The plot of the observed rate constants versus the ADP concentration was fit by a hyperbola to give **k**_**-AD**_ and **K**_**AD**_. The error bars report the standard deviation for three independent experiments (see Table 2).

We determined the extent of ADP inhibition for the ATP-induced actomyosin dissociation to estimate both the ADP affinity and the ADP release rates of the actomyosin complex for the WT protein and the functionally and kinetically most affected K265E mutant. We titrated the ATP-induced (2 mM) actomyosin dissociation reaction with ADP over a broad range of concentrations (0–1000 µM) and followed the dissociation reaction by the reduction in light scattering, which followed single exponential kinetics (Fig. 10a). The observed ADP-induced reduction of the dissociation rate was fitted with a hyperbola to give the ADP affinity of the actomyosin complex **K**_**AD**_ and the ADP release rate from actomyosin **k**_**−AD**_ (Fig. 10b, Table 2). We found significant changes for the K265E mutant, where the ADP affinity increased from 73.3 ± 7 to 40.7 ± 8 µM.

### Alanine and Glutamic Acid substitutions alleviate the inhibitory effect of PBP, whilst the exchange of Lysine 265 to Glutamine has no effect

**Figure 11:**
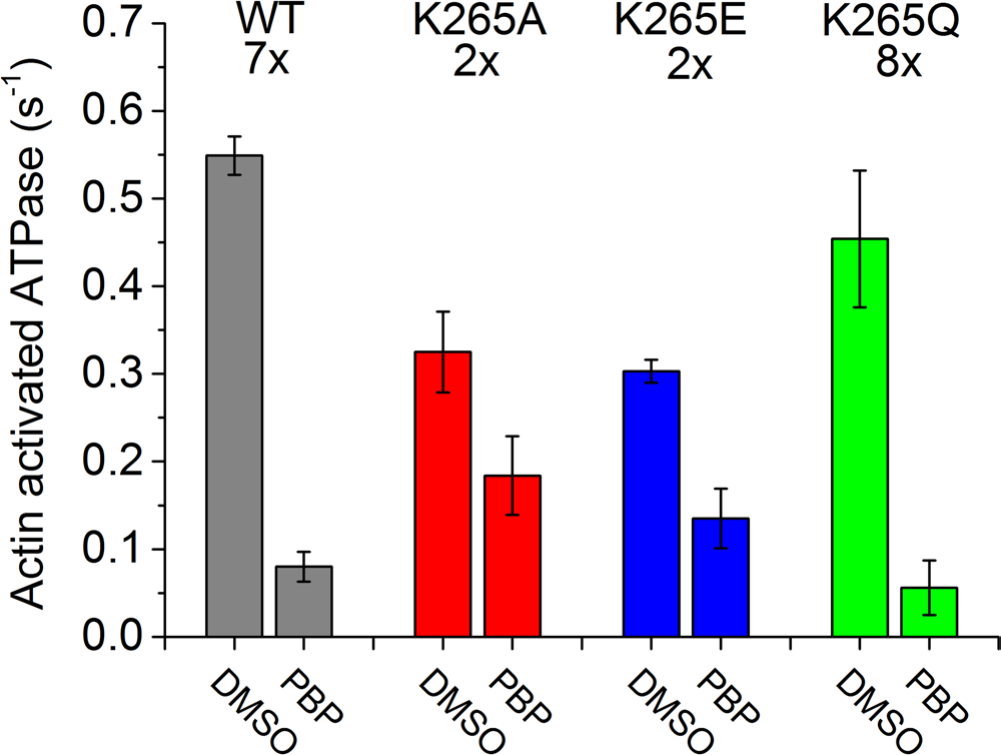
Pentabromopseudilin-mediated inhibition of steady-state ATPase activities for WT and mutant constructs. Inhibition profile of the actin-activated ATPase activity (20 µM F-actin) of WT and mutant myosins by 100 µM PBP. Numbers on top indicate the extent of inhibition (n-fold change) compared to the 5% DMSO control. Data are mean values of 3–5 independent measurements and error bars give the standard deviation. The differences between DMSO control and PBP were significant in all cases (p < 0.05).

Finally, as PBP is proposed to allosterically inhibit myosin-2 via a K265 mediated interaction, we wanted to evaluate the influence of the mutations on its inhibitory potential. As such, we performed steady-state ATPase assays for the WT and mutant myosins at a concentration of 20 µM F-actin in the absence and presence of 100 µM PBP (Fig. 11). Under these conditions, the ATP turnover of the WT construct is 7-fold reduced from 0.55 to 0.08 s^−1^. This result is in agreement with published data^9^. Mutant constructs K265E and K265A were inhibited 2-fold, whereas for K265Q, the inhibitory effect was 8-fold.

## Discussion

In the present study, we show that the evolutionary conserved amino acid residue lysine-265 in *Dd* myosin-2 is implicated in the kinetic and mechanical coupling of nucleotide- and actin-binding sites in the motor domain. To elucidate the impact of changes in the size and charge of the side chain at position 265, we analysed the kinetic and functional properties of three mutant myosin motor constructs in direct comparison with the WT construct M765-2R. We chose to make use of the truncated motor domain construct with an artificial lever arm (M765-2R) for this study to guarantee comparability with the published work on *D. discoideum* myosin-2 kinetics and motor function, and studies on the effect of the pseudilin class of myosin inhibitors. To further allow for a high degree of consistency between our data and the literature, we performed steady-state ATPase assays and *in vitro* motility assays under similar conditions, which are in line with published data (25 °C, 25 mM KCl, pH 7.3). The transient kinetic experiments have been performed under different conditions (20 °C, 100 mM KCl, pH 7.0), which are the accepted standard parameters for stopped-flow experiments where myosin is stabilised in the absence of actin^15, 18–21, 27, 28^.

**Figure 12:**
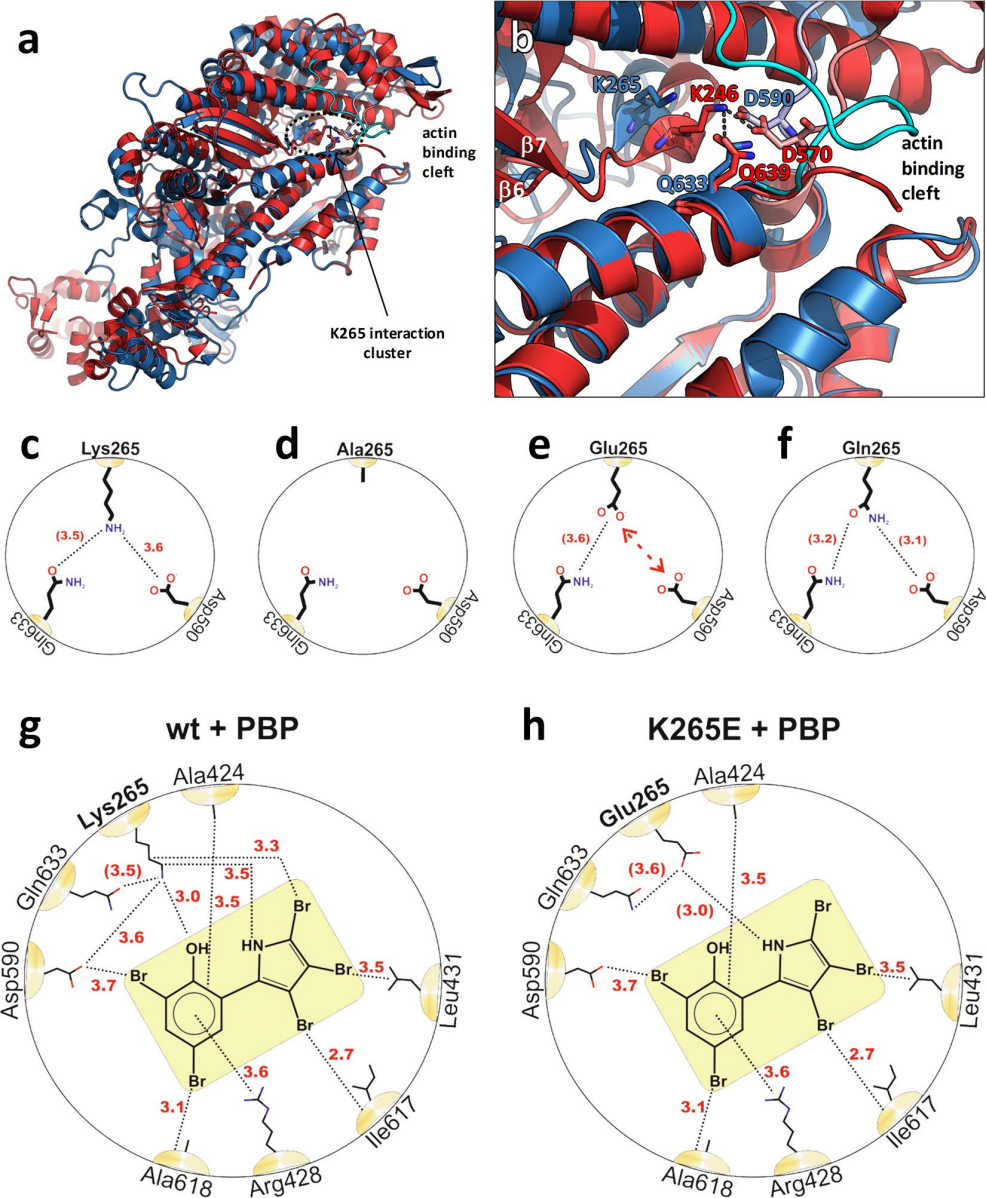
Structural model for the molecular basis of lysine-265 mutation-mediated effects on the allosteric pathway. (**a**) Overview picture of the myosin motor domain depicting the location of K265 in the rigor-like structures of *Dd* myosin-2 (blue, pdb: 2AKA)^23^ and of *G. gallus* myosin-5a (red, pdb: 1OE9)^24^. Note the completely closed actin-binding cleft in the myosin-5a structure and the partially closed cleft in myosin-2, which is proposed to close completely upon binding to F-actin. (**b**) Close-up view of the actin-binding cleft near K265 (*Dd* myosin-2) and K246 (*Gg* myosin-5a). (**c**) Schematic representation of the allosteric branching point K265/D590/Q633. The K265-Q633 interaction is established in the rigor state with closed actin-binding cleft. Red numbers indicate interaction distances in Å. The numbers in parentheses are distances proposed for the position of Gln633 in *Dd* myosin-2 with completely closed actin binding cleft (based on myosin-5a, pdb: 1OE9). (**d–f**) Illustration of the effects of mutations K265A, K265Q and K265E. Note the complete absence of interactions for Ala265, the repulsion between Glu265 and Asp590 and the retained Gln633 interaction of Gln265. (**g**) Schematic representation of interactions in the PBP binding pocket of WT *Dd* myosin-2 (based on pdb: 2JHR)^9^. (**h**) Mutation K265E eliminates specific interactions and thereby weakens the interaction network of PBP.

Here, we discuss the structural basis of the lysine-265 interaction pattern based on the crystal structures of *Dd* myosin-2 and *G. gallus* myosin-5a and relate it to our kinetic and functional analysis to develop a model for the proposed allosteric pathway. In the myosin-5a rigor state, lysine-246 can interact with two amino acids, glutamine Q639 and aspartic acid D570 (Fig. 11a,b). The rigor-like structure of myosin-5a is a good model for the strongly actin-bound state, as it is assumed that in the presence of F-actin the actin-binding cleft of *Dd* myosin-2 also closes completely^29^. Accordingly, direct interactions between K265 and Q633, as well as D590, are expected to be enabled for myosin-2 in the presence of F-actin (Fig. 12b,c). K265 is located four residues C-terminal to the seventh strand (β7) of the central beta-sheet. β7 in turn is linked to switch-1 via β6. Q633 and D590 are part of the L50 subdomain and the strut, respectively. The L50 subdomain forms the major part of the actin-binding site and the strut loop (Fig. 12b, shown in salmon and light blue colour for myosin-5a and myosin-2, respectively) has an impact on the conformation of the cleft^30–32^. This loop connects the upper and lower 50 K domain together with loop-2, thereby contributing to cleft-closure during actin binding. Q639 (*Gg* myosin-5a) and Q633 (*Dd* myosin-2) are located at the tip of helix W, which marks the transition to loop-2, the major actin-binding element of myosin (loop-2 is shown in cyan for *Dd* myosin-2 and is not resolved in the crystal structure of *Gg* myosin-5). Importantly, helix W itself also forms part of the direct electrostatic interaction surface with actin^30^. Furthermore, the base of helix W is linked to β3, which in turn anchors the converter domain to the core motor domain via the SH1 and SH2 helices. As such, disturbances of these amino acid interaction patterns cause conformational changes in the cleft. Cleft closure caused by F-actin binding plays an important role in the activation of myosin. Conformational changes at this site impact the twisting of the central seven-stranded beta-sheet, which is directly related to the nucleotide binding site. By this pathway F-actin activates myosin’s ATPase activity^33, 34^.

Of the three mutants tested, substitution of the positively charged lysine-265 with an uncharged and small alanine residue resulted in an intermediate phenotype. Here, we observed a reduction in motility, unaffected basal ATPase rate, a reduction in the actin activated ATPase rate, no effect on actin binding, a slight reduction in the affinity to actin and attenuation of the inhibitory effect of PBP. Based on our structural analysis, the effects observed in this mutant are likely due to the interactions with D590 and Q633 being abolished (Fig. 12d). Here, conformational changes caused by F-actin binding cannot effectively be relayed to the nucleotide-binding site via the central beta-sheet, disturbing F-actin-mediated activation.

The K265E mutant displayed the strongest phenotype, with all parameters except for the basal ATPase rate being reduced compared to the WT myosin. The strongly increased basal ATPase and P_i_ release rate, reduced ATP hydrolysis rate and 2-fold higher affinity for ATP are associated with the weak binding states, and were not observed in the K265A mutant. We propose that these effects are due to the repulsive interaction of the negatively charged glutamic acid (E265) and the negative charge of the aspartic acid, D590 (Fig. 12e, red double arrow). Furthermore, the observed reduction of the inhibitory potency of PBP can be rationalized by the elimination of specific interactions compared to the WT (Fig. 12g,h).

With only a moderate increase in the basal ATPase rate and slight decrease in actin affinity, the K265Q mutant’s kinetic and functional profile is largely comparable to that of the WT myosin. In this mutant, we exchanged the positively charged lysine with an uncharged glutamine, which has a side chain of similar size. In contrast to the K265A mutant, glutamine can still interact with the aspartic acid (D590) in the strut and does not interfere with the actin-binding cleft closure (Fig. 12f). Additionally, the interaction with the L50 subdomain is unperturbed in this mutant, which can be observed as an unchanged or even slightly increased F-actin activation.

### Structural and mechanistic model of the proposed allosteric pathway

Based on the crystal structures of *Dd* myosin-2 and of *G. gallus* myosin-5a in their rigor-like state, we postulate a regulatory branch in the amino acid interaction pattern K265-D590-Q633. In this pattern, two key mechanisms of allosteric regulation in the catalytic head domain can be suggested.

The first mechanism is proposed to regulate the effect of F-actin in the strong binding states of myosin by the K265-Q633 interaction. This interaction is abolished in the K265A mutant, as the alanine residue is small and uncharged. Therefore, it can be assumed that Q633 is not stabilized in a defined position as it is with an intact K265-Q633 interaction. In the K265E mutant, the positively charged lysine is exchanged by a negatively charged glutamic acid. Because of its terminal amino group, Q633 could be attracted by E265 resulting in a change of position. Exchanging K265 with a glutamine in the K265Q mutant removes the positive charge from the amino acid side chain. Therefore, charge-based interactions between the two terminal amino groups are not present in this mutant.

The negative effect on F-actin sensitivity in the K265A and K265E mutants indicates a change in the loop-2 conformation, as Q633 is located at the tip of helix W, which directly connects to loop-2 (Fig. 12b). In the absence of interactions, or with interactions that build up conformational tension on amino acid side chains K265 and Q633 by electrostatic attraction (Fig. 12c), the activating effect of F-actin binding is reduced. The positive effect on the F-actin sensitivity in the K265Q mutant suggests that even in the WT, the effect of F-actin binding is attenuated to a certain extent by the interaction between K265 and Q633. This can be assumed to have an overall regulatory role in keeping myosin from excessive F-actin-mediated activity.

The second mechanism appears to regulate the weak binding states by the K265-D590 interaction. Whilst the deletion of the interaction between amino acid side chain K265 and D590 in the K265A mutant causes no effect (Fig. 12d), the K265E mutation has an impact on the weak binding states. We expect, that exchanging the positively charged lysine by a negatively charged glutamic acid causes a repulsive reaction with D590 (Fig. 12e). In the K265Q mutant, this charge-based repulsion is absent (Fig. 12f).

In the WT, the interaction of a positively charged lysine and a negatively charged aspartic acid can be assumed to cause strain on the peptide backbone. This could have an impact on the torsion angle and the conformation in general. Inserting a negatively charged glutamic acid or a non-charged glutamine at position 265 can reduce the strain or, by repulsion from D590, apply conformational stress on the amino acid 265 and tension on the peptide backbone surrounding position 265. The expected change in the torsion angle could explain the effects on the weak binding states via the proposed allosteric communication pathway.

Analysis of the mutation’s influence on the inhibitory potential of pentabromopseudilin supports the hypothesis that this amino acid is pivotal in mediating the effect of the bound inhibitor to the nucleotide- and actin-binding sites (Fig. 12g,h). The fact that the mutants K265E and K265A are severely attenuated in their response to the bound inhibitor, whereas the mutant K265Q could sense and relay the conformational information, additionally underlines the central role of K265 in the proposed communication pathway^9–11^. This knowledge can help to further optimize the potential of inhibitors, which in part act via this pathway, like the pseudilin inhibitors of mammalian myosin-1 and myosin-5^9–11^ or the recently described *Fusarium* myosin-1 and myosin-5 inhibitors^12–14^.

In summary, these findings lead us to postulate a regulatory branch in the conserved amino acid pattern K265-D590-Q633. This evolutionarily highly conserved regulatory branch appears to be important to allosterically modulate myosin in its basal and F-actin-activated states, and is therefore crucial for the overall function of myosin.

## Methods

### Reagents

Standard chemicals were purchased from Sigma-Aldrich; restriction enzymes, polymerases and DNA-modifying enzymes were purchased from Thermo Fisher Scientific.

### Plasmid Construction

The expression vectors used for the production of mutant myosin-2 constructs were based on pDXA-3H and were created as described earlier^16^. The point mutations were introduced in M765-2R, a fusion construct comprising the first 765 residues of the *Dd* mhcA gene linked to codon 264 extending to 505 of the *Dd* α-actinin gene. All constructs were tagged at their C-terminus with the peptide Asp-Ala-Leu-(His)_8_. Plasmids encoding M765-2R with K265 mutations were created by site-directed mutagenesis. The oligonucleotides used to PCR the myosin mutants were the following: K265A (5′ TCAATCCTACCTTTTAGAG*GCC*TCACGTGTCGTTTTCCAATC), K265E (5′ TCAATCCTACCTTTTAGAG*GAA*TCACGTGTCGTTTTCCAATC), and K265Q (5′ TCAATCCTACCTTTTAGAG*CAA*TCACGTGTCGTTTTCCAATC) with the mutated residues in *italics* and the reverse primer K265rev (5′ ATTGAAGCACCACTAATGAAACCAGCACTGTTGAATTG) used for all three constructs. All myosin constructs were confirmed by sequencing.

### Protein Production and Purification

The plasmids for the WT and the three mutants of the *Dd* myosin-2 construct with artificial lever arm and a C-terminal (His)_8_-tag (M765-2R) were transformed into AX3-Orf^+^ cells by electroporation as described earlier^15^. Transformants were grown at 21 °C in HL-5c medium and selected in the presence of 10 µg/ml G418 and 100 units/ml penicillin/streptomycin. Screening for the production of the mutated myosin constructs and protein purification was performed as described^17^. In brief, after cell lysis the suspension was subjected to high-speed centrifugation for 1 h at 4 °C and 60,000 rpm (Beckman 70 Ti rotor, 265,000 × g) in the absence of ATP and a second time in the presence of ATP to make use of the reversible interaction of myosin with actin. The myosin-enriched precleared supernatant was sterile filtered and applied to a pre-equilibrated Ni^2+^-nitrilotriacetic acid (NiNTA) column. The protein was washed with low salt and high salt buffers exactly as in (Manstein and Hunt, 1995)^17^ and eluted using a linear gradient of Imidazole from 30 mM to 1000 mM. The WT and mutant proteins typically eluted at an Imidazole concentration of approx. 200 mM. Fractions containing the pure protein (>95% purity) were combined and dialysed over night to remove the Imidazole. The protein was concentrated (Vivaspin concentrators, 50 kDa molecular weight cut-off), supplemented with either 3% (for kinetic studies) or 30% (for direct functional assays) sucrose, flash-frozen in liquid nitrogen and stored at −80 °C. Rabbit skeletal muscle actin was purified as described by Lehrer and Kerwar^35^. Pyrene-labelled actin was prepared as described by Criddle *et al*.^36^.

### *In vitro* motility Assays

Actin-sliding motility was measured at 25 °C as described previously^16^. The myosin constructs were specifically immobilised on nitrocellulose-coated glass surface decorated with anti-His_5_ antibody (mouse monoclonal IgG_1_, Qiagen) via their C-terminal His_8_ tags. The assay buffer AB (25 mM imidazole, pH 7.3; 25 mM KCl; 4 mM MgCl_2_; 1 mM EGTA; 5 mg/ml Glucose; 0.1 mg/ml Glucose-Oxidase; 0.02 mg/ml Catalase) was supplemented with 4 mM ATP to initiate filament sliding. The movement of TRITC-phalloidin-labelled actin filaments on the myosin lawn was recorded for each individual *Dd* myosin-2 construct. Automated actin filament tracking was performed with the program DiaTrack 3.05 (Semasopht, Switzerland). Data analysis was performed with Origin 2016 (Originlab, USA).

### Kinetic Measurements

ATP turnover was measured at 25 °C in assay buffer (25 mM HEPES [4-(2-hydroxyethyl)-piperazine-1-ethane sulfonic acid] (pH 7.3), 25 mM KCl, 5 mM MgCl_2_, 1 mM DTT, 1 mM ATP,) using a NADH(reduced nicotinamide adenine dinucleotide)-coupled assay as described previously^19^. To determine the inhibitory effect of PBP, ATPase assays were performed in the presence of 20 µM F-actin and either 5% DMSO (control) or 100 µM PBP^9^ (in the presence of 5% DMSO). Transient kinetic experiments were performed at 20 °C with either a Hi-tech Scientific SF-61 single mixing or double mixing stopped-flow system (TgK Scientific Limited, Bradford on Avon, U.K.) in assay buffer (20 mM MOPS [3-(N-Morpholino)-propanesulfonic acid] (pH 7.0), 100 mM KCl, 5 mM MgCl_2_, 1 mM DTT) using procedures and kinetic models described previously (Fig. 3 and references)^27, 37^. Fluorescence traces were averaged from three to six experiments and single exponential fits were performed to obtain *k*_*obs*_ values. Error bars represent standard deviation. Mant-nucleotides (Jena Bioscience, Germany) were excited at 365 nm and detected after passing through a KV 389 nm cut-off filter. Tryptophan fluorescence was excited at 297 nm and detected using a WG 320 nm cut-off filter. Phosphate release was measured using MDCCPBP (N-[2-(1-maleimidyl)ethyl]-7-(diethylamino)coumarin-3-carboxamide fused to phosphate binding protein; obtained from Life Technologies, Carlsbad, CA) as described previously^3, 38^. Coumarin fluorescence was excited at 430 nm and detected using a 455 nm cut-off-filter.

### Statistical analyses

Unless otherwise stated, data are reported as mean ± standard deviation. Student’s paired t test (2-tailed) was applied to determine the significance of the differences in ATPase activity or sliding velocity between WT and mutant myosin or in the absence or presence of inhibitor, respectively. Statistical significance is assigned as ns for P > 0.05, *for P ≤ 0.05, **for P ≤ 0.005, ***for P ≤ 0.0005.

### Data Availability

The datasets generated during and/or analysed during the current study are available from the corresponding author on reasonable request.
